# The Associative Pattern Between Segmental Arterial Damage and Complete Neurological Disorder After Spinal Cord Injury: A Case-Control Study

**DOI:** 10.7759/cureus.35918

**Published:** 2023-03-08

**Authors:** Baptiste Boukebous, Lorenzo Serfaty, Te Ra R Hodges-Tai, Joseph F Baker, Jean Denis Moyer, Marc Antoine Rousseau

**Affiliations:** 1 Orthopaedics and Traumatology, Waikato District Health Board, Hamilton, NZL; 2 Orthopedics and Traumatology, Bichat–Claude Bernard Hospital, Paris, FRA; 3 Orthopedics and Traumatology, Waikato District Health Board, Hamilton, NZL; 4 Intensive Care Unit, Beaujon Hospital, Clichy, FRA; 5 Orthopaedics and Traumatology, Bichat–Claude Bernard Hospital, Paris, FRA

**Keywords:** blood supply, medullar blood flow, cord ischemia, vasculature surrounding the spinal column, segmental artery, spinal cord injury

## Abstract

Introduction: The prevalence of vascular trauma surrounding the thoracic spine following Spinal Cord Injury (SCI) is unknown. The potential for neurologic recovery is uncertain in many cases; in some cases, neurologic assessment is not possible, for example, in severe head injury or early intubation, and detection of segmental artery injury may help as a predictive factor.

Objective: To assess the prevalence of segmental vessel disruption in two groups, with and without neurologic deficit.

Material and methods: This is a retrospective cohort study, with a group SCI American Spinal Injury Association (ASIA) E and a group SCI ASIA A. All patients had a high-energy thoracic or thoracolumbar fracture from T1 to L1. Patients were matched 1:1 (one ASIA A matched with one ASIA E) according to the fracture type, age, and level. The primary variable was the assessment of the presence/disruption of the segmental arteries, bilaterally, around the fracture. Analysis was performed twice by two independent surgeons in a blinded fashion.

Results: Both groups had 2 type A, 8 type B, and 4 type C fractures. The right segmental artery was detected in 14/14 (100%) of the patients with ASIA E and in 3/14 (21%) or 2/14 (14%) of the patients with ASIA A, according to the observers, p=0.001. The left segmental artery was detectable in 13/14 (93%) or 14/14 (100%) of the patients ASIA E and in 3/14 (21%) of the patients ASIA A for both observers. All in all, 13/14 of the patients with ASIA A had at least one segmental artery undetectable. The sensibility varied between 78%to 92%, and the specificity from 82% to 100%. The Kappa Score varied between 0.55 and 0.78.

Conclusion: Segmental arteries disruption was common in the group ASIA A. This may help to predict the neurological status of patients with no complete neurological assessment or potential for recovery post-injury.

## Introduction

Vascular trauma around the spinal column has been well identified at the cervical level, contributing to spinal cord injury (SCI). The assessment for possible vertebral artery dissection is common [1]. At the thoracic and thoracolumbar levels, traumatic lesions of the vasculature surrounding the spinal column have been subject to several case reports, but their prevalence and clinical consequences remain uncertain. The dissipation and energy absorption during trauma are well identified in contributing to soft tissue injury [2]. A better description of the vascular damage after SCI and a better knowledge of their associative pattern with complete neurological disorders may constitute a tool to help clinicians assess patients sedated with unknown neurological status. Failure to obtain a complete initial neurological assessment may occur between 10 to 30% of cases [3-5]. Patients may be sedated because of hemodynamic instability, a brain injury with a poor Glasgow score, or confusion. The causative relationship between the vascular insult and the onset of the neurological disorder remains unclear. Experimental ligation of thoracic segmental arteries has resulted in paraplegia in animal models involving felines, canines, and swine [6-9].

The ligations caused a decrease in the medullar blood flow and consequent cord ischemia. Some segmental arteries branch in anterior radiculomedullary arteries, which supply the anterior spinal artery (ASA) [10-12]. The terminal perforating capillaries from the ASA supply the grey matter's anterior horn and the white matter's anteromedial part, forming the vertical medullary network [10]. Unlike animal models, the human anatomy is characterized by a substantial degeneration of the anterior radiculomedullary arteries during embryologic development; their number decreases from 31 pairs to less than 10 arteries, mostly unilaterally on the left side [10,13]. At the thoracic level, the vascular supply of the human spine relies, on average, on two radiculomedullary arteries, and below, the Adamkiewicz artery (AKA) supplies the thoracolumbar hinge. A horizontal medullary arterial network arises from each segmental artery, and the terminal capillaries supply the white matter. The terminal capillaries from the horizontal and vertical arterial networks irrigate two distinct territories, and the area between these territories is vascularized by diffusion, so exceedingly sensitive to ischemia [10,13,14]. The anatomical description of the blood supply network demonstrates that the thoracic and thoracolumbar spinal cord highly depends upon the segmental arteries at each level.

This study assessed the prevalence of segmental artery disruption in two groups of patients with a vertebral fracture at the thoracic and thoracolumbar cord level, with and without neurologic deficit. We hypothesized that the disruption of segmental arteries is more frequent in the group with a deficit.

## Materials and methods

This paper complied with the Strengthening the Reporting of Observational Studies in Epidemiology (STROBE) guidelines.

Study design and setting

To address the hypothesis, we conducted a retrospective cohort study with two groups: a group of patients with SCI ASIA E (American Spinal Cord Injury Association) [15] and a group of patients with SCI ASIA A. The patients were recruited in two level-1 trauma centers. The period of inclusion was from 2016 and 2022.

Participants and study size

Only the thoracic and thoracolumbar medullar cord fractures were considered, from T1 to L1. Patients with multilevel fractures were not included (except adjacent fractures). All the patients sustained high-energy trauma, and patients with pathologic or low-energy fractures were excluded.

High energy was an inherent characteristic of the group ASIA A; all the patients suffered from polytrauma. All the patients underwent operative spinal fracture management, with at least posterior instrumentation. All the patients were initially admitted to the critical care unit and had a trauma CT scan (vertex to the proximal femur) with arterial contrast for the initial screening. The quality of this CT scanner was a key factor, as the detection of the segmental arteries was based on it. The arterial contrast had to be perfectly realized (with no venous contrast), and the slice thickness had to be at </= 1mm without movement artifact. The patients for whom these quality criteria were not met were excluded.

The patients for the group ASIA A were recruited first; there were more eligible patients for the group ASIA E, so a selection was made to obtain a 1:1 matching based on the type of fracture first, then the age and level. The type of fracture was determined per the AO Spine Injury Classification System [16]. The type A fractures were compression injuries, the type B fractures were distractions and type C translations. A total of 14 patients were selected in the group ASIA A and matching complete from a larger cohort of patients classed ASIA E.

Variables and bias

**Figure 1:**
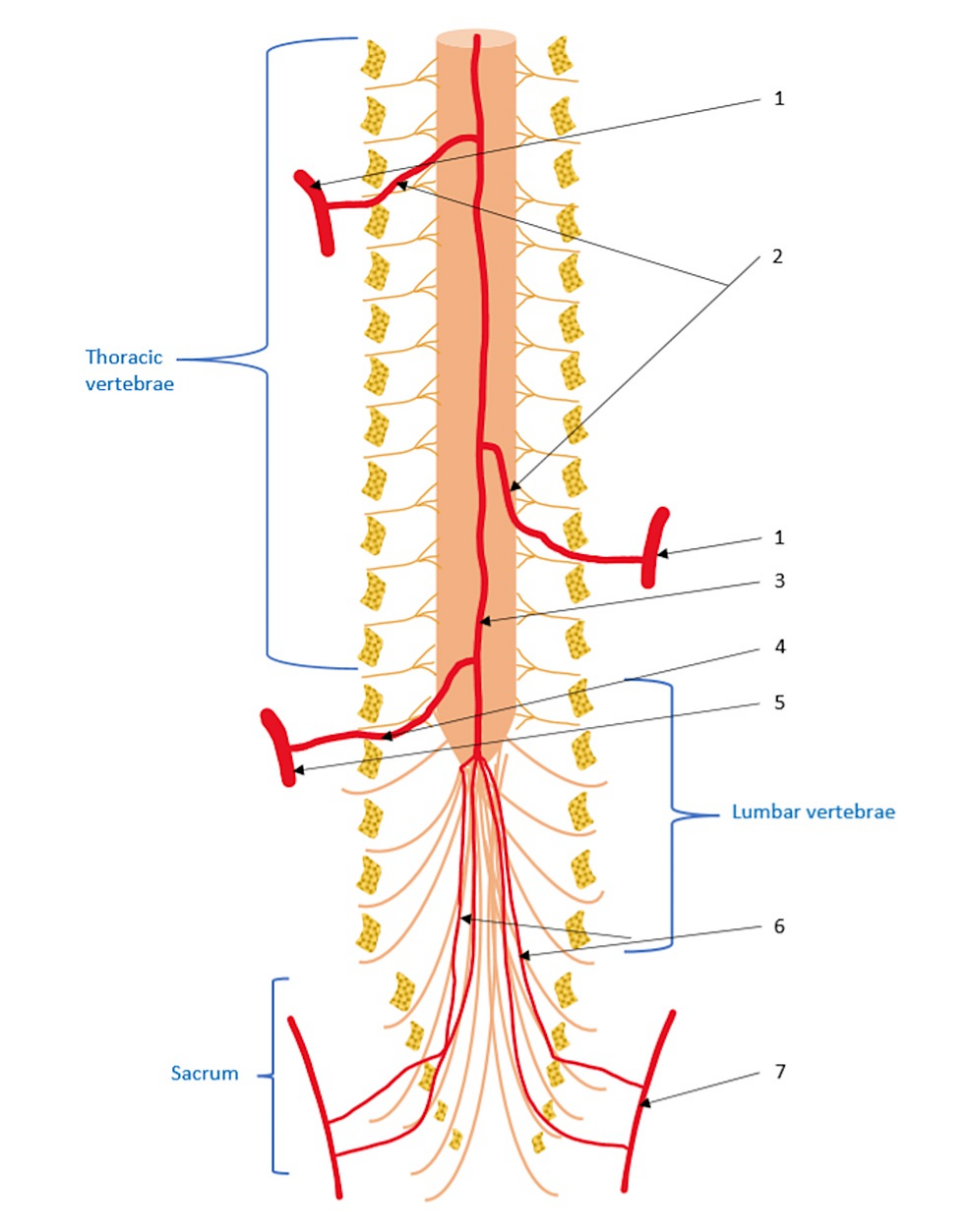
Vascularization of the spine with two radiculomedullary arteries at the thoracic level. 1: thoracic segmental artery; 2: anterior radiculomedullary arteries; 3: anterior spinal artery (ASA); 4: Adamkiewicz artery; 5: lumbar segmental arteries; 6: ponytail arteries; 7: sacral arteries. Source: drawing from the authors.

The primary variable was the assessment of the segmental arteries around the fracture. At each level, these vessels arise from the aorta bilaterally and then cross alongside the middle-height side of each vertebral body towards the posterior wall (Figure 1). Because the aorta is at the anterior and left aspect of the vertebral body, the left segmental arteries are shorter than the right-the segmental arteries branch in a spinal artery, a posterior cutaneous artery, and an intercostal artery.

The segmental arteries were sought at the fracture level and the two adjacent levels, with multiplanar reconstructions (MRP) of CT images (PACS software Kodak/Carestream Health®, Rochester, NY, USA). The vessels were followed up to the posterior intercostal branches and sought in the axial, sagittal, and coronal planes. In some cases, the artery was complexly visible at the level fracture because it wedged into the fracture but reappeared posteriorly, branching in a posterior intercostal artery (Figure 3). Three-dimensional video reconstructions were made for illustration purposes only. The images were analyzed twice by independent surgeons in a blinded fashion.

Cord compression is a well-known factor for developing neurological deficits due to spine dislocation or impingement. This parameter was measured as the ratio between the anteroposterior diameter of the canal at the narrowest level around the fracture and the normal anteroposterior diameter immediately adjacent to the fracture.

Other variables collected were the mechanism of injury, the other associated lesions, and the neurological status at the last follow-up.

Statistical methods

The main outcome was to compare the occurrence of segmental artery disruption in the two groups; a non-parametric Fisher test was used. The sensibility and specificity of the absence of one of the two segmental arteries were calculated. The inter-individual agreement between the two surgeons was assessed by measuring Cohen's Kappa score.

A multivariable regression was then performed in which the neurological status (ASIA A or E) was the outcome. The associations between ASIA and the arterial disruption, the compression, the gender, and the level were tested with Wilcoxon tests, Spearman correlations, or a single variable Generalized Linear Model (for categorical variables). Each variable associated with ASIA with a p-value less than 0.2 were selected for the multivariable regression. The data management and statistical calculations were performed using R Software (R 3.4.3). The risk alpha was set at 0.05.

Ethical approval

This study was conducted following the ethical standards of the 1964 Declaration of Helsinki. Ethical approval was not required according to the MR-004 reference methodology; the study was registered in the Commission nationale de l'informatique et des libertés database register (n°2226771).

## Results

Participant and descriptive data

The two groups had similar ages (31.3 years and 31.7 years for groups ASIA A and ASIA E, respectively, p=0.99). There were 2 type A fractures, 8 type B, and 4 type C fractures in both two groups. There were 5/14 (36%) women in the group ASIA A and 3/14 (21%) in the group ASIA E (p=0.67). The complete characteristics of the cohort are detailed in table 1.

**Table 1:** Demographic data ASIA: American Spinal Injury Association

No	Sex	Age	AO type	Level	ASIA	ASIA LFU	Type of accident	Other traumatic lesions	Traumatic laminectomy	Canal narrowing	Left Segmental	Right Segmental
1	F	45	A	T11	A	A	High-speed car accident	Lung contusion, rib fractures, pelvic hematoma, femoral shaft fracture	no	40%	1	0
2	M	36	B	T12	A	A	High-speed car accident	-	no	54%	1	1
3	F	25	C	T6	A	A	High-speed car accident	Brain injury, hepatic laceration	no	52%	0	1
4	F	28	C	L1	A	A	Defenestration	Pelvic injury, hepatic laceration, rib fractures	no	52%	0	0
5	F	23	C	T8	A	A	Defenestration	Rib fractures, hemo-pneumoperitoneum	yes	80%	0	0
6	M	32	B	T7	A	A	High-speed motorbike accident	Pneumothorax	no	22%	0	0
7	M	50	B	T6	A	A	Bike accident	Brain injury, hemo-pneumoperitoneum, sternum fracture	yes	51%	0	0
8	M	20	B	T12	A	A	High-speed car accident	Brain injury	no	90%	0	0
9	M	29	C	T9	A	A	Collision with a car	Hemothorax	no	15%	0	1
10	M	52	B	T4	A	A	Accidental fall from a 5-meter height	Flail chest	no	12%	0	0
11	M	27	C	L1	A	A	Defenestration	Pelvis fracture, tibial plateau fracture, bilateral calcaneus fracture	no	97%	0	0
12	M	25	B	L1	A	A	Collision with a car	Pelvic fracture, femoral shaft fracture, hemoperitoneum	no	28%	0	0
13	F	19	B	T12	A	A	High-speed motorbike accident	Pneumothorax	yes	30%	0	1
14	M	28	A	T12	A	A	High-speed motorbike accident	Brain contusion, pneumothorax	no	50%	1*	1
15	M	33	B	T11	E	E	High-speed motorbike accident	Brain contusion	yes	0%	1	1
16	M	47	B	T10	E	E	High-speed car accident	Rib fractures	yes	25%	1	1
17	M	46	A	T12	E	E	Accidental fall from a 4-meter height	Hemothorax	yes	46%	1	1
18	M	27	B	T8	E	E	High-speed car accident	Splenic injury, brain, and face injury	no	0%	1	1
19	M	22	B	T11	E	E	High-speed motorbike accident	Hemothorax, hepatic lacerations	yes	0%	1	1
20	M	55	C	T9	E	E	High-speed motorbike accident	-	yes	0%	1	1
21	M	18	C	T8	E	E	Truck accident	Pneumothorax	yes	0%	1	1
22	F	19	A	T12	E	E	High-speed motorbike accident	Brain contusion, pneumothorax	yes	28%	1	1
23	F	16	B	T12	E	E	High-speed car accident	Facial injury	no	0%	1	1
24	M	40	C	T9	E	E	High-speed motorbike accident	Haemopneumothorax, retroperitoneal hematoma, splenic injury, pelvic fractures, aortic pseudo aneurysm	yes	0%	1	1
25	M	48	C	T7	E	E	High-speed car accident	Pneumothorax	yes	0%	1	1
26	F	33	B	T8	E	E	High-speed motorbike accident	Sternum fracture, lung contusion	no	15%	1	1
27	M	16	B	T6	E	E	High-speed motorbike accident	Pneumothorax, brain injury	yes	42%	1	1
28	M	19	B	T8	E	E	High-speed car accident	Mediastinal hematoma, brain injury	yes	0%	1	1

Main outcome

**Figure 2:**
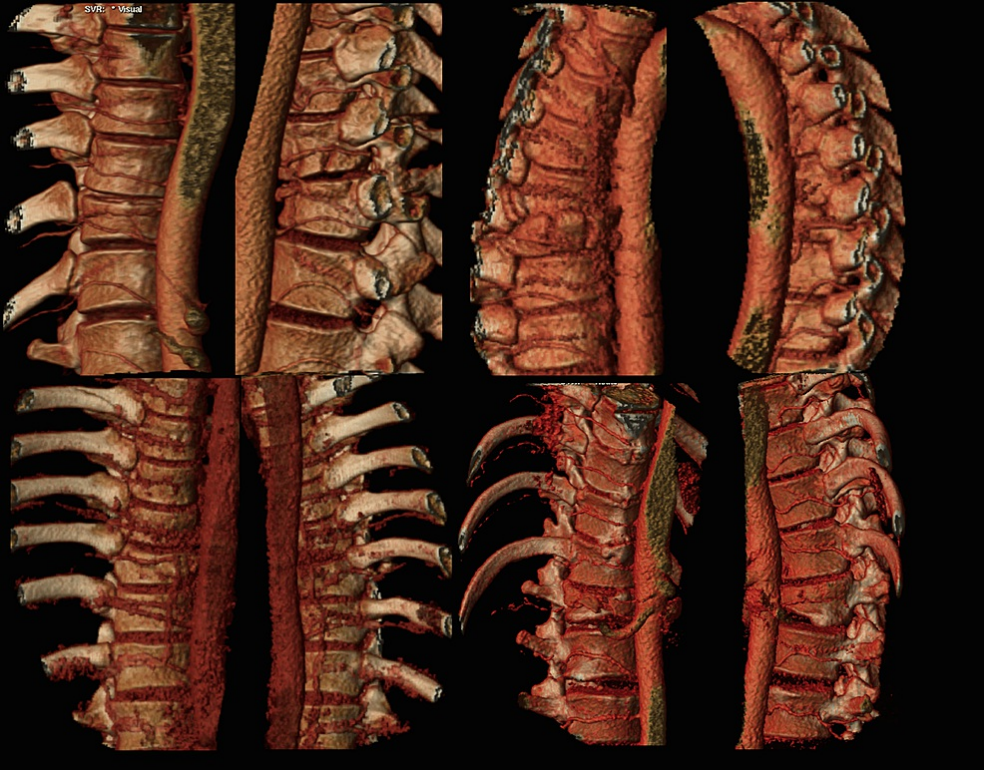
No segmental artery disruption. 3D reconstructions of arterial phases. All the segmental arteries are visible next to the vertebral bodies: Posteriorly, the segmental arteries branch into posterior intercostal arteries. For the bottom right case, the right segmental artery is hardly perceptible because it is wedged into the fracture; the artery reappears posteriorly while branching in a posterior intercostal artery.

**Figure 3:**
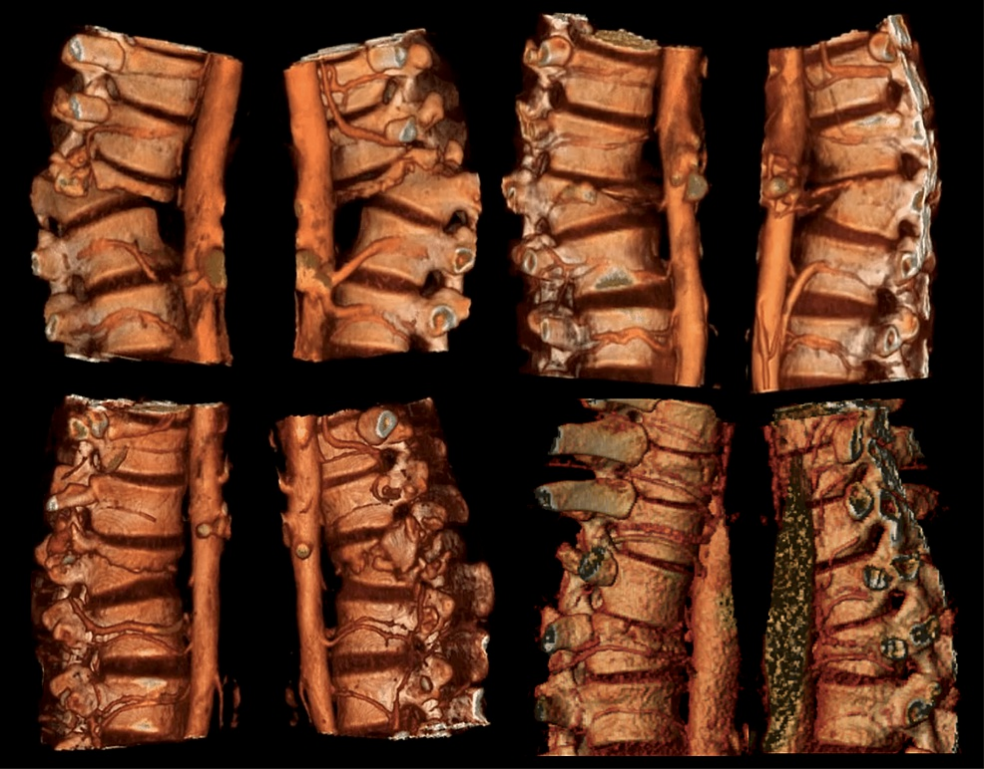
Segmental artery disruption 3D reconstructions of arterial phases. The segmental arteries at the fracture level are not discernible.

**Figure 4:**
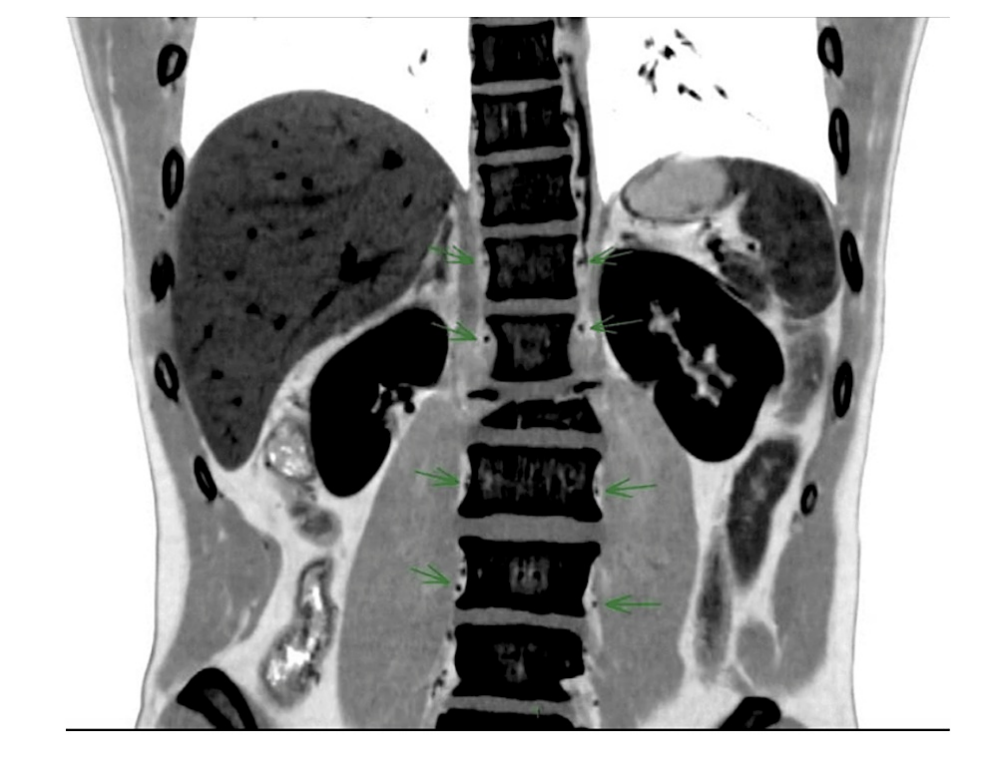
Coronal reconstruction, patient ASIA A. The contrast was reversed to improve visibility. All the segmental arteries are visible next to the vertebral bodies (green arrows), except at the fracture level.

The right segmental artery was detected in 14/14 (100%) of the patients with ASIA E (figure 2) by both observers, and in 3/14 (21%) or 2/14 (14%) of the patients with ASIA A (figure 3, 4, Video 1), according to the observers, p=0.001. One patient of the group ASIA A had both segmental arteries at the level of the fracture, but the segmental on the right, above the fracture, was absent (Figure 4). The left segmental artery was detectable in 13/14 (93%) or 14/14 (100%) of the patients ASIA E, according to the different observers, and in 3/14 (21%) of the patients ASIA A, for both observers, p<10-4. Three patients in the group ASIA A also had a non-detectable left segmental artery at the level above the fracture.

**Video 1:** Reconstructions around the fractures, segmental artery disruption. 3D reconstructions of arterial phases, deleting all soft tissues except arteries and vertebrae, on both sides of fractures, two vertebrae above, two vertebrae below. Aorta is the great vessel located in the left front side of vertebrae, from which segmental arteries arise. In each case, reconstruction failed to detect the segmental arteries on both sides of the fractures, whiles these vessels were well identified at the other levels.

Considering 56 observations (detection in both sides in both groups), the sensibility varied between 78% and 82%, according to the observers, and the specificity was 96% for both observers. Cohen's Kappa score was 0.55 (moderate agreement), with an agreement of 78%.

All in all, 13/14 of the patients with ASIA A had at least one segmental artery undetectable (video 2). Considering 28 observations (presence or absence of a segmental artery on either side), the sensibility was 92% for both observers, and the specificity varied from 92% to 100%, according to the observers. Cohen's Kappa score was 0.78 (substantial agreement), with an agreement of 89%.

**Video 2:** Reconstructions around the fractures, segmental artery disruption

The mean canal narrowing was 29.9%, 48.8% in the group ASIA and 11.1% in the group ASIA E (p=0.0002). The occurrence of lamina fractures was lower for ASIA A: 2/14 (14%) versus 11/14 (78%) (p=0.003). The segmental artery disruption and the canal compression were significantly associated with ASIA level in the multivariable regression (p<10-4 and p=0.03, respectively).

## Discussion

The main result was that among patients with the presumably same type of trauma and the same type of fracture, the one presenting a disruption of the segmental vessels on at least one side of the fracture was more likely to suffer from a complete neurological disorder. This feature had good specificity, sensibility, and inter-observer reliability.

This study has several limitations. First, the results demonstrate a robust associative pattern, but the causative relationship between vascular disruption and neurological disorder remains questionable. The matching between the two groups aimed to select patients with similar seriousness of trauma, but it is likely that patients ASIA A had more local tissue trauma. For instance, in whiplash injury, some fractures would have been more displaced at the time of injury before 'recoiling' to the position seen on imaging. To demonstrate a causative association, it would have been necessary to include patients with intermediary neurological status (ASIA B, C, and D), which is complex and probably requires the joint effort of several Level 1 trauma centers. Second, the CT is not accurate enough to evaluate intra-canal vascularization. There is no information about the ASA, the AKA, and the posterior intra-canal vascularization. The development of a compensatory collateral network has been described lately after segmental ligation [17]; it is still unknown if such mechanisms are involved acutely after SCI. The venous network has not been assessed, and a similar disruption of the segmental veins can be expected; moreover, venous thrombosis is known to lead to cord ischemia [18]. Lastly, there is no MRI imaging to assess the cord. Although genuine transection of the cord is exceedingly rare [19], other factors contributing to the neurological disorder may have been overlooked, such as intrathecal hematoma.

The associative pattern between the initial ASIA status and vascular insult is a new finding that can be employed in daily clinical practice. The unspecified initial neurological status is frequent, up to 28%, for numerous reasons, including early sedation and brain injury. For those patients, detecting a segmental artery disruption would indicate a severe neurological impairment with a high degree of certainty. 

Several authors have described experimental paraplegia by ligation of segmented arteries in animal models: Marsala et al. [7], Tsitsopoulos et al. [8] in felines, Etz et al. in swine [6], Kato et al. in canines [9]. In humans, there is abundant literature on the risks associated with Adamkiewitz artery ligation during spinal surgery, leading to severe neurological impairment. This risk is rare, ranging from 0% to 0.75% [20,21]. Nonetheless, these studies focus on programmed surgical interventions, not traumatic situations, which is a significant bias. In elective procedures, the hemodynamic is optimally maintained throughout the anesthesia, and patients do not have multi-location failures, so the medullar blood flow is preserved.

Vascular deficits are probably underdiagnosed and underappreciated in SCI at the thoracic and thoracolumbar levels. We propose classifying the damage into three categories, from the larger vessels to the micro-vascularization. The aorta represents the largest vessel, the segmental arteries represent the intermediary vascularization, and the micro vascularization corresponds to the microcapillary supply into the canal. At the largest scale, Santoro et al. reported 42 cases of blunt traumatic injuries [3]; vascular lesions could be full-thickness laceration, obstruction, intimal tear, or pseudoaneurysm of the aorta. Similarly, vertebral artery dissection may occur after cervical spine trauma, and Salkov et al. demonstrated the association between macrovascular damage and the severity of neurologic deficit after cervical trauma [22]. Thirty-six percent of cases reported by Santoro et al. suffered a neurologic deficit, and two notable differences stand out compared to our series. Santoro et al.'s mortality rate was 30% due to vascular damage. At the same time, none of our ASIA A group patients died because of a vascular cause, and the aorta was intact. Secondly, 70% of the cases had type C fractures in their series, while in ours, mainly type B. All this suggests that vascular damage after SCI likely has various patterns, mechanisms, and clinical consequences. The segmental artery lesions appear less life-threatening but result in a more significant neurologic deficit. The reasons for the vascular disruption remain unclear, but several hypotheses can be drawn: blunt injury with avulsion, compressed or wedged by the fracture fragments, dissection, or thrombosis. Due to an intravertebral cleft, wedged segmental arteries have been described following osteoporotic fractures [23]. 

**Figure 5:**
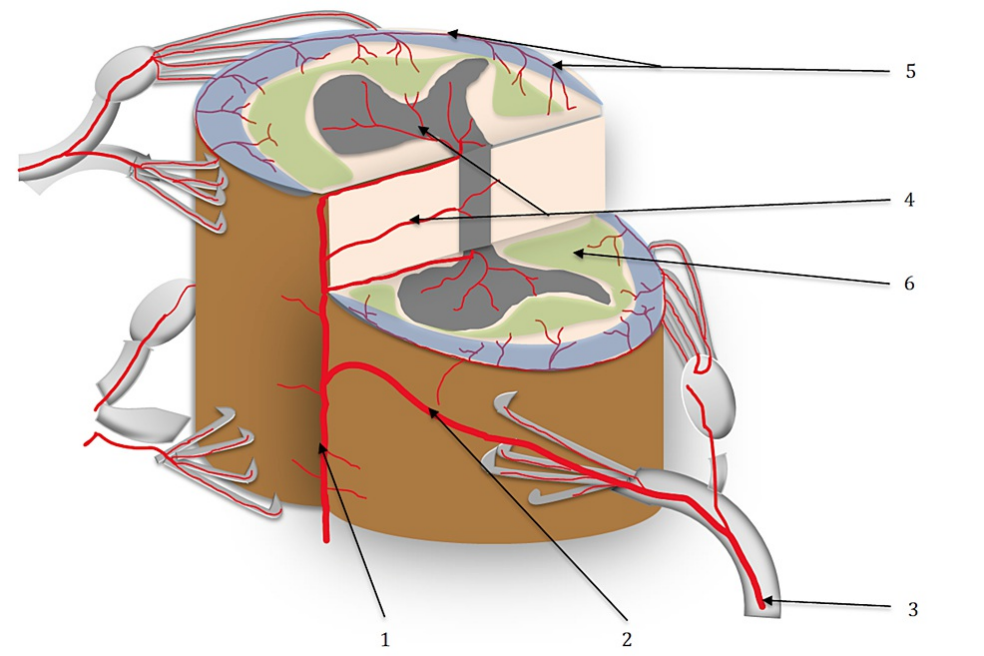
watershed zones 1: anterior spinal artery; 2: radiculomedullary artery; 3: segmental artery; 4: vertical network; 5: horizontal network; 6: watershed zones. Source: drawing from authors.

At the microscopic scale, Tator et al. reported histological findings of arterial and venous thrombosis in traumatized human spine sections [24]. An inflammatory cascade involving hyperemia leads to an ischemic environment several times after the trauma. Ultimately, the healing process produces scar tissues, so the marrow loses its histological shape [24-26]. Our study suggests that local ischemia might also result from segmental vessel injuries. If the level injured is the level of the AKA or another to the anterior radiculomedullary artery, a disruption of the flow of the ASA is expected. We did not assess the location of the AKA because of the limitation of the imaging technique; however, 8/14 patients in the ASIA A group had a fracture location around the thoracolumbar hinge, which is the location of the AKA. In any case, at each level, the disruption of the segmental arteries can alter the horizontal vascular crown network of the cord. The border area between these two territories, called "watershed zones," is fragile because of the junction between the terminal systems (Figure 5). The watershed zones are significantly sensitive to arterial hypotension and are at risk for hypo-perfusion ischemia [13,14]. In the brain, similar watershed zones are explicitly involved in the pathophysiology of strokes [17]. The posterior network of the spine is significantly more developed than the anterior; each segmental artery branches in a posterior radiculomedullary artery, destined to one posterior spinal artery on each side. This posterior network, richer in supply, is less impacted by the disruption of the segmental arteries.

This study brings perspectives on the management of SCI. First, even if this study is not designed to demonstrate a causative relationship between segmental vessel disruption and ischemia, it emphasizes the need for protection against the secondary injury cascade to decrease the ischemic stress [27]. Not only could the disruption of the segmental vessels be a tool for clinicians for their initial neurological assessment, but it also may be a prognosis factor for potential recovery post-injury. There still needs to be precise data about the benefits of emergent surgical decompression, compared to delayed surgery, for patients with complete SCI [28]. However, our study suggests the segmental arteries could be wedged into or compressed by the fracture, so the acute distraction of the fracture, to remove the extrinsic compression is a theory that deserves further investigation. Finally, the segmental arteries are accessible via endovascular procedures [29]. Endovascular angioplasty could be attempted for patients with disrupted segmental arteries.

## Conclusions

Disruption of the segmental arteries was overwhelmingly associated with patients with severe SCI. This associative pattern is a helpful feature that may help clinicians in their early neurological assessment, especially in patients sedated, and constitute a prognosis factor for potential recovery post-injury.

Because of the thoracic and thoracolumbar cord's poor vascular supply and watershed zones, such disruption can theoretically be implicated in decreased medullar blood flow. This study calls for further investigations to assess a possible causative relationship between vascular insult, ischemia, and neurological consequence.
